# The Association between Socio-Demographic Characteristics and Using Pain Assessment Tools among Critically Ill Patients

**DOI:** 10.3390/medicina59040759

**Published:** 2023-04-13

**Authors:** Mohammad Rababa, Shatha Al-Sabbah, Anwar M. Eyadat, Hanan A. Abusbaitan

**Affiliations:** 1 Adult Health Nursing Department, Jordan University of Science and Technology, Irbid 22110, Jordan; 2 Department of Community and Mental Health, Faculty of Nursing, Jordan University of Science and Technology, Irbid 22110, Jordan

**Keywords:** pain, critically ill patients, demographics, assessment tools

## Abstract

*Background and Objectives*: Pain is still undertreated among ICU patients, especially cognitively impaired patients. Nurses play a crucial role in their management. However, previous studies found that nurses had insufficient knowledge about pain assessment and management. Some nurses’ socio-demographic characteristics, such as being female; age; years of experience; type of unit, either medical or surgical; education level; years of nursing experience; qualification; position; and hospital level, were found to be associated with their practices of pain assessment and management. This study aimed to examine the association between nurses’ socio-demographic characteristics and the use of pain assessment tools for critically ill patients. *Materials and Methods*: A convenience sample of 200 Jordanian nurses responded to the Pain Assessment and Management for the Critically Ill questionnaire to achieve the study’s aim. *Results*: The type of hospital, academic qualification, years of experience as a critical care nurse, and hospital affiliation were significantly associated with increased use of self-report pain assessment tools for verbal patients, while the type of hospital and hospital affiliation was significantly associated with an increased use of observational pain assessment tools for nonverbal patients. *Conclusion*: Examining the association between socio-demographic characteristics and the use of pain assessment tools for critically ill patients is essential for quality pain practice.

## 1. Introduction

Pain is a crucial problem in critically ill patients [1]. Critically ill patients feel pain due to implicit illness or harm during their stay in intensive care units (ICUs); such pain ranges between moderate to severe at rest and during routine surgical or other medical procedures [2]. Several studies have been conducted worldwide to assess pain prevalence among critically ill patients. In India, a prospective observational study found that the prevalence of moderate-to-severe pain among cancer patients admitted to the ICU is 75.40% [3]. Another Brazilian study found that the incidence of acute pain in critically ill patients is 23.6% [4]. In Jordan, approximately 33% of critically ill patients in the ICU suffered pain at rest, and 10% experienced moderate-to-severe pain [5]. Another Jordanian study showed that 58% of patients experienced pain when admitted to ICUs [6].

Uncontrolled pain places patients at a higher risk for adverse psychological and physiological consequences that may be life-threatening [7]. Untreated pain leads to long-term psychological distress, acute neurohumoral changes, and neuronal remodeling. Moreover, uncontrolled pain may increase the risk of developing chronic pain syndrome and impact the patient’s functioning, quality of life, and long-term well-being status [8], causing sympathetic activation, secreting inflammatory mediators, acute kidney injury, and potential organ failure [4].

Pain is still undertreated among ICU patients, especially cognitively impaired patients [9]. Given that pain is a significant problem among critically ill adult patients, nurses play a crucial role in its management. However, previous studies found that nurses had insufficient knowledge about pain assessment and management [10,11]. This lack of knowledge was related to nurses’ heavy workload, lack of education regarding pain assessment tools, poor documentation and communication of pain assessment priorities [10], and negative attitudes toward pain management [12]. On the other hand, some nurses’ socio-demographic characteristics, such as being female; age; years of experience; type of unit, i.e., either medical or surgical; education levels [13,14,15]; years of nursing experience; qualification; position; and hospital level [16], were found to be associated with nurses’ practices of pain assessment and management [13,14].

Pain assessment is still a key measure among critically ill ICU patients and should be performed as early as possible. Among ICUs patients, such as those who are cognitively impaired, intubated, or with dementia or communication deficits, the pain remains unrelieved and undertreated due to the inability to self-report. Nurses still feel it is somewhat unimportant to assess pain among cognitively dis-intact patients compared to those who are cognitively intact [9]. Self-reported and observational pain scales cannot be used interchangeably because of the fluctuations in critically ill patients’ mental status [17]. Observational pain scales such as the Behavioral Pain Scale (BPS), the Critical-Care Pain Observation Tool (CPOT), pupillometry, family caregivers’ help, and vital signs’ fluctuations/changes are recommended for pain assessment among cognitively impaired patients [18]. In comparison, the pain assessment scale (FPAS) was significantly correlated with the numeric pain rating scale and visual analog scale in cognitively intact patients, specifically those older than 40, who preferred this FPAS [19]. Despite the variations in pain assessment tools, nurses play a critical role in ensuring proper and efficient pain assessment and management.

Based on the evidence, there is a gap in knowledge regarding the association between nurses’ socio-demographic characteristics and using pain assessment tools globally. This study will help bridge this gap to see where future services in Jordan could be developed to better suit critically ill patients’ needs. The study’s results will generate principal evidence of the necessity of assessing and managing pain and developing interventions to increase awareness and knowledge and improve practices regarding pain assessment and management among nurses. Based on our knowledge, this is the first study conducted in Jordan to assess the relationship between nurses’ socio-demographic characteristics and using pain assessment tools. The study aimed to examine the association between nurses’ socio-demographic characteristics and the use of pain assessment tools for critically ill patients in Jordan.

## 2. Materials and Methods

This is a descriptive correlational study. The researchers selected three Jordanian hospitals (teaching, public, and private) according to their convenience for this study to recruit a convenience sample of 200 registered nurses who are working in ICU settings with a minimum clinical experience of 6 months. The teaching hospital has a total capacity of 750 beds, with three ICUs for adults: a critical care unit (CCU), a cardiac intensive care unit (CICU), and an intermediate ICU. The public hospital has a total capacity of 400 beds, with two ICUs for adults: a critical care unit (CCU) and a cardiac intensive care unit (CICU). In contrast, the private hospital has a total capacity of 150 beds, with one ICU for adults: a cardiac intensive care unit (CICU). After calculating the required sample size based on the G*Power analysis, which used a power of 0.80 and a medium effect size of 0.25, the recruited sample was sufficient for yielding significant statistical analyses and controlling for incomplete data.

The Institutional Review Board (IRB) department of Jordan University of Science and Technology approved this study. Written consent was obtained from the participants before data collection. Voluntary participation was maintained throughout the whole procedure of data collection, as well as the confidentiality and privacy of collected data. The participants were informed that they could withdraw from the study at any time. No personal information about the participants was disclosed to anyone.

The use of pain assessment tools among critically ill patients was measured using the four items of the Pain Assessment and Management for the Critically Ill questionnaire developed by Rose et al. [20]. The four items used in this study are related to pain assessment among verbal (two items) and nonverbal critically ill patients (two items). Regarding the use of pain assessment tools for verbal critically ill patients, the nurses were categorized into the “Yes” or “No” group according to self-report responses on the relevant item in the questionnaire: “do you use a self-report pain assessment tool for patients who are able to communicate?” Then the nurses selected a specific self-report tool that they used for verbal patients from multiple choices.

Regarding the use of pain assessment tools for nonverbal critically ill patients, the nurses were categorized into the “Yes” or “No” group according to self-report responses on the relevant item in the questionnaire: “do you use an observational pain assessment tool for patients unable to communicate?” Then the nurses selected a specific observational pain tool that they used for nonverbal patients from multiple choices.

The English version of the questionnaire was used in the study since English is the primary language of nursing instruction in Jordanian hospitals. Permission to reuse the instrument was granted from the developers [20]. The questionnaire items had a Cronbach alpha reliability score of 0.77.

The demographic characteristics of participants, including gender, age, academic qualification, years of clinical experience, usual shift rotation, employment status, and ICU primary specialty, were collected by a self-administered demographic questionnaire filled out by the nurses.

After obtaining the IRB approval, the researchers agreed with the administrators of the selected hospitals on the dates/times for data collection according to the potential participants’ work schedules. The researcher sent an invitation email to participate to all potential participants after getting a list of their emails and discussing their eligibility with their administrators. The researchers met with all nurses who responded to the invitation email in a private and quiet room in a group of 20 to briefly describe the study’s promise and get their written consent. The nurses who gave their consent were handed a folder containing the study questionnaire and asked to drop it at the reception counter of their work department when they finished. The average time required to fill out the questionnaire was 10 min. A week later, the researcher came back to collect the completed questionnaires. The data were collected in May 2022.

SPSS version 25.0 was used to analyze the data related to the study purpose. Percentages and frequency were used to describe the proportion of nurses using the pain assessment tools for their verbal and nonverbal patients. Post hoc analysis involved pairwise comparisons, using multiple z-tests with a Bonferroni correction to examine the difference in the frequency of pain assessment tools for verbal and nonverbal patients between independent nurse proportions. Binomial logistic regression analysis was performed to determine the significant predictors of using pain assessment tools for verbal and nonverbal patients.

## 3. Results

### 3.1. Nurses’ Clinical Characteristics

The participating nurses in the present study had an average age of 27.24 ± 3.66 years. Most of the nurses were males, holding a bachelor’s degree, working as a registered nurse for five years and above, and working in a teaching hospital. The details of nurses’ socio-demographic characteristics are outlined in Table 1.

### 3.2. Nurses’ Clinical Characteristics and Using Pain Assessment Tools for Verbal Patients

The post hoc analysis involved pairwise comparisons using multiple z-tests of two proportions with a Bonferroni correction (Table 2) shows statistically significant differences in the proportion of nurses using pain assessment tools for patients who are able to self-report symptoms according to the type of hospital, hospital affiliation, academic qualifications, and years of experience as a critical care nurse. The proportion of nurses working in private hospitals who use pain assessment tools for their patients who are able to self-report was significantly higher than that for those working at public or university hospitals (*p* = 0.022).

Regarding academic qualification, the proportion of nurses with a bachelor’s degree who used the pain assessment tool in patients who are able to report was statistically significantly (*p* ≤ 0.001) higher than that of nurses with a diploma or master’s degree. In terms of the years of experience as a critical care nurse, the proportion of nurses who had a clinical experience of five years or less and used the pain assessment tool in patients who are able to report was statistically significantly higher than that of nurses with experience for 5–10 years or more than 10 years (*p* ≤ 0.001). Regarding hospital affiliation, the proportion of nurses affiliated with a teaching hospital and used the pain assessment tool in patients who are able to report was statistically significantly lower than that of nurses affiliated with a large community or moderate community hospital (*p* = 0.013). Other variables, including employment status, usual shift rotation, number of ICU, number of beds in the ICU, and years of experience as a registered nurse, were not significantly associated with using pain assessment tools for patients who are able to report (*p* ≥ 0.05).

### 3.3. Nurses’ Clinical Characteristics and Using Pain Assessment Tools for Nonverbal Patients

The proportion of nurses using pain assessment tools for patients who are unable to self-report symptoms was significantly different according to the type of hospital and hospital affiliations (Table 3). The proportion of nurses working at private hospitals who used pain assessment tools for patients who are unable to self-report pain was significantly higher than that of nurses working at public or university hospitals (*p* < 0.001). Regarding hospital affiliation, the proportion of nurses who worked at a teaching hospital and used the pain assessment tool in patients who are unable to report was statistically significantly lower than that of nurses with a large community of ≥200 beds or a moderate community of 50–199 beds (*p* < 0.001).

Other variables, including qualification, employment status, usual shift rotation, number of ICU, number of beds in the ICU, years of experience as a registered nurse, and years of experience as a critical care nurse, were not significantly associated with the use of the pain assessment tool for patients who are unable to report.

### 3.4. Predictors of Using Pain Assessment Tools

A binomial logistic regression analysis was performed to ascertain the effects of qualifications, years of experience as a critical care nurse, type of hospital, and hospital affiliation on the likelihood that participants using pain assessment tools for patients who are able to self-report. The logistic regression model was statistically significant, χ^2^(8) = 29.53, *p* < 0.0005. The model explained 29.4% (Nagelkerke R2) of the variance in using pain assessment tools and correctly classified 90.5% of cases. Of the four predictor variables, the experience as a critical care nurse was a statistically significant predictor (Table 4). Nurses with experience as critical care nurses for less than five years had 1.917 times higher odds of using the pain assessment tool than nurses with experience of 5–10 years. Nurses with a diploma had 1.382 lower odds of using pain assessment tools than nurses with a bachelor’s degree.

A binomial logistic regression was also performed to ascertain the effects of qualifications, years of experience as a critical care nurse, type of hospital, and hospital affiliation on the likelihood that participants used pain assessment tools for patients who are unable to self-report. The logistic regression model was statistically significant, χ^2^(4) = 28.872, *p* < 0.0005. The model explained 30.5% (Nagelkerke R2) of the variance in using pain assessment tools and correctly classified 87.45% of cases. Only one of the two predictor variables was statistically significant (Table 5). Nurses working in teaching hospitals had 2.44 times lower odds of using the pain assessment tool for patients who are unable to self-report than those working in a large community with ≥200 beds.

## 4. Discussion

Our study found that various nurses’ socio-demographic characteristics influenced their practice toward pain among critically ill patients. Consistently, previous studies found significant differences between nurses working in private, public, and university-affiliated hospitals regarding the level of knowledge and practice, academic and clinical qualifications, job satisfaction, and patient-to-nurse ratio, which may affect pain quality of care [21,22]. Moreover, consistent with our findings, Hamdan’s [23] study found a significant association between the type of hospital and the use of pain assessment tools for patients who are able and unable to self-report symptoms. It also reported that a lower proportion of nurses who used pain assessment tools were working in governmental hospitals, similar to our study findings. However, Hamdan [23] reported that nurses in university-affiliated hospitals used pain assessment tools more often than nurses in other hospitals. Our study found that most nurses who used pain assessment tools worked in private hospitals. Our findings were supported by the finding of a recent study [9] that private-hospital and recently graduated nurses are more skilled in pain assessment methods than senior nurses working in public hospitals.

Conversely, recent studies [24,25] showed no significant relationship between hospital type and the use of pain assessment tools by nurses or physicians. This finding could be explained by the fact that public hospitals are most likely to have nurses with high burnout and patient–nurse rates [26]. Hospital affiliations could also affect patients’ quality of pain care [27]. In terms of the use of pain assessment tools for patients who are able and unable to self-report, our study showed that the proportion of nurses who worked in a teaching hospital and used the pain assessment tool in patients who are able and unable to self-report was statistically significantly lower than of nurses working in large community (≥200 beds) and moderate community (50–199 beds) hospitals. In our study, nurses working in the teaching hospital were found to be a predictor for the underuse of pain assessment tools for patients who are unable to self-report. A study by Zhang et al. [28] supported similar findings in which nurses working in teaching hospitals had poor attitudes and knowledge regarding pain assessment tools.

Several studies revealed that higher nurses’ experience levels facilitated adequate pain evaluation and treatment among critical care patients [29,30,31,32]. However, the current study found that nurses with experience of 5 years or less used the pain assessment tool in patients who are able to report significantly more often than nurses with experience of more than five years. Consistent with these results, Hamdan [23] and Rose et al. [20] reported that nurses with experience of 5 years or less used pain assessment tools for both being able and unable to self-report more often than experienced nurses. The suggested explanation for these findings is that nurses with fewer years of experience were more interested in updating themselves with the most recent pain assessment guidelines, emphasizing the importance of using pain assessment tools [23].

In the current study, the proportions of nurses using pain assessment tools for verbal and nonverbal critically ill patients seem to be equal. However, nurses using observational tools for assessing pain behaviors in nonverbal patients may misunderstand or misinterpret these behaviors [33]. The behaviors include withdrawal, facial grimacing, negative vocalizations, and restless behaviors. Even though nurses use pain assessment tools for nonverbal patients, they do not clearly understand how pain manifests in those patients since they cannot effectively articulate their comfort needs [33].

### 4.1. Implications for Nursing Education, Practice and Research

The study findings could be utilized to develop continuing education programs for nurses on the use of pain assessment tools for critically ill patients. Moreover, nursing scholars may benefit from the findings to create course syllabi related to pain assessment practice for critically ill patients. Moreover, the findings could contribute to changing pain practice policies in Jordanian hospitals to be tailored to the special comfort needs and challenges of critically ill patients, especially those who are unable to self-report symptoms. Further examinations of the issue of underassessment of pain in critically ill patients and how it relates to unrelieved pain among those patient groups are recommended.

### 4.2. Limitations

The current study has some limitations. The current study employed a descriptive correlational design, which fails to establish a causal relationship among the studied variables. The current study’s findings cannot be generalized to all nurses from all clinical departments since the recruited nurses are working in ICUs. Using a convenience sampling method to recruit the nurses in this study may be associated with a selection bias and threaten the internal validity of the study’s findings. Furthermore, using a self-reported questionnaire may be associated with recall bias and measurement errors. The study measured the nurses’ self-reported responses on using a pain assessment tools questionnaire, not the accuracy of using pain scales or the action taken if pain scores were high. Furthermore, the unequal sample sizes in the comparison groups may impact the results of some comparisons. The results of the t-test need to be interpreted within the context of the unequal comparison group sizes, and this may induce bias and limit the generalizability of the results. Future studies should consider a larger sample size and well-controlled research designs and statistical models. Moreover, this study evaluated the use of specific pain scales and not potential other strategies for pain assessment. More controlled and multidisciplinary research, using experimental designs, larger samples, and multisite settings, is needed to overcome the abovementioned limitations.

## 5. Conclusions

The present study examined the association between nurses’ socio-demographic characteristics and their use of pain assessment tools for verbal and nonverbal critically ill patients. The type of hospital, academic qualification, years of experience as a critical care nurse, and hospital affiliation were significantly associated with an increased use of self-report pain assessment tools for patients who are able to self-report. In contrast, hospital affiliation was significantly associated with an increased use of observational pain assessment tools for patients who are unable to self-report. The nurses’ related factors, such as their demographic and professional characteristics and how they relate to the problem of delayed management of pain among critically ill patients, especially those unable to self-report symptoms, need future examinations to reach optimal pain management.

## Figures and Tables

**Table 1:** Socio-demographic characteristics of the study sample (N = 200).

Characteristics	Number	%
Gender		
Male	120	60%
Female	80	40%
Qualifications		
Diploma	21	10.5
BScN/BN	152	76.0
Masters	27	13.5
Employment status		
Full-time	138	69.0
Part-time	43	21.5
Casual	19	9.5
Type of Hospital		
Public	78	39.0
Private	84	42.0
Teaching	38	19.0
Years of experience as a registered nurse		
˂5	90	45.0
5–10	86	43.0
>10	24	12.0

BScN/BN: Bachelor of Science in Nursing/Bachelor’s in Nursing.

**Table 2:** The association between nurses’ clinical characteristics and using pain assessment tools for patients who are able to self-report.

Variables	Use of Pain Assessment Tool for Able Patients		*p*-Value
	No N (%)	Yes N (%)	
Type of hospital			0.022
Public	13 (68.4)	65 (35.9)	
Private	4 (21.1)	80 (44.2)	
University	2 (10.5)	36 (19.9)	
Academic qualifications			<0.001
Diploma	7 (36.8)	14 (7.7)	
BScN/BN	8 (42.1)	144 (79.6)	
Masters	4 (21.1)	23 (12.7)	
Employment status			0.317
Full-time	15 (78.9)	123 (68.0)	
Part-time	4 (21.1)	39 (21.5)	
Casual	0 (0.0)	19 (10.5)	
Usual shift rotation			0.501
Days only	3 (15.8)	18 (9.9)	
Evenings only	2 (10.5)	46 (25.4)	
Nights only	3 (15.8)	25 (13.8)	
Rotating shifts	11 (57.9)	92 (50.8)	
Number of ICUs			0.299
1–2	17 (89.5)	144 (79.6)	
more than 2	2 (10.5)	37 (20.4)	
ICU beds			0.102
≤25 beds	16 (84.2)	119 (65.7)	
>25 beds	3 (15.8)	62 (34.3)	
Hospital affiliation			0.013
Teaching	3 (15.8)	9 (5.0)	
Large community ≥200 beds	5 (26.3)	106 (58.6)	
Moderate community 50–199 beds	11 (57.9)	66 (36.5)	
Years of experience as a registered nurse			0.586
<5	10 (52.6)	80 (44.2)	
5–10	8 (42.1)	78 (43.1)	
>10	1 (5.3)	23 (12.7)	
Years of experience as a critical care nurse			0.003
<5	5 (26.3)	119 (65.7)	
5–10	11 (57.9)	49 (27.1)	
>10	3 (15.8)	13 (7.2)	

BScN/BN, Bachelor of Science in Nursing/Bachelor’s in Nursing; ICU, intensive care unit.

**Table 3:** The association between nurses’ clinical characteristics and using pain assessment tools for patients who are unable to self-report.

Variables	Use of Pain Assessment for Unable Patients		*p*-Value
	No N (%)	Yes N (%)	
Type of hospital			<0.001
Public	14 (82.4)	64 (35.0)	
Private	1 (5.9)	83 (45.4)	
University	2 (11.7)	36 (19.6)	
Academic Qualifications			0.400
Diploma	1 (5.9)	20 (10.9)	
BScN/BN	12 (70.6)	140 (76.5)	
Masters	4 (23.5)	23 (12.5)	
Employment status			0.062
Full-time	16 (94.1)	122 (66.7)	
Part-time	1 (5.9)	42 (22.9)	
Casual	0 (0.0)	19 (10.4)	
Usual shift rotation			0.656
Days only	2 (11.8)	19 (10.4)	
Evenings only	3 (17.6)	45 (24.6)	
Nights only	4 (23.5)	24 (13.1)	
Rotating shifts	8 (47.1)	95 (51.9)	
Number of ICUs			0.400
1–2	15 (88.2)	146 (79.8)	
>2	2 (11.8)	37 (20.2)	
ICU beds			0.172
≤25	14 (82.4)	121 (66.1)	
>25	3 (17.6)	62 (33.9)	
Hospital affiliation			<0.001
Teaching	7 (41.2)	5 (2.8)	
Large community ≥200 beds	5 (29.4)	106 (57.9)	
Moderate community 50–199 beds	5 (29.4)	72 (39.3)	
Years of experience as a registered nurse			0.359
<5	6 (35.3)	84 (45.9)	
5–10	10 (58.8)	76 (41.5)	
>10	1 (5.9)	23 (12.6)	
Years of experience as a critical care nurse			0.067
<5	8 (47.1)	116 (63.4)	
5–10	9 (52.9)	51 (27.9)	
>10	0 (0.0)	16 (8.7)	

BScN/BN, Bachelor of Science in Nursing/Bachelor’s in Nursing; ICU, intensive care unit.

**Table 4:** Predictors of using pain assessment tools for patients who are able to self-report.

Variables	B	S.E.	Wald	*p*	Exp(B)	95% C.I. for EXP(B)	
						Lower	Upper
Academic qualifications			4.711	0.010			
Diploma	−1.382	0.821	1.014	0.092	0.251	0.050	1.255
BScN/BN	0.756	0.707	0.960	0.284	2.131	0.533	8.510
Years of experience as a critical care nurse			0.640	0.035			
˂5	1.917	0.952	0.000	0.044	6.799	1.052	43.960
5–10	0.369	0.854	0.00	0.666	1.446	0.271	7.715
Type of hospital			1.125	0.999			
Public	−0.046	1.065	1.116	0.965	0.955	0.118	7.701
Private	−0.016	1.097	0.323	0.988	0.984	0.115	8.442
Hospital affiliation			9.849	0.124			
Teaching	−0.956	0.862	8.998	0.267	0.384	0.071	2.082
Large community ≥200 beds	1.109	0.733	0.051	0.131	3.030	0.720	12.757

B, beta coefficient; S.E., standard deviation; *p*, *p*-value; Exp(B), odds ratio; BScN/BN, Bachelor of Science in Nursing/Bachelor’s in Nursing; ICU, intensive care unit.

**Table 5:** Predictors of using pain assessment tools for a patient who is unable to self-report.

Variables	B	S.E.	Wald	*p*	Exp(B)	95% C.I. for EXP(B)	
						Lower	Upper
Type of hospital			4.366	0.113			
Public	−0.709	0.970	0.534	0.965	0.492	0.0.073	3.295
Private	1.561	1.286	1.474	0.988	4.764	0.383	59.229
Hospital affiliation			12.123	0.002			
Teaching	−2.441	0.759	10.34	0.001	0.087	0.020	0.386
Large community ≥200 beds	0.077	0.775	0.010	0.921	1.080	0.236	4.930

B, beta coefficient; S.E., standard deviation; *p*, *p*-value; Exp(B), odds ratio.

## Data Availability

The data can be obtained from the corresponding author upon request.
